# Global clinical trial activity in metastatic castration-resistant prostate cancer from January 2020 to March 2026: a landscape analysis

**DOI:** 10.3389/fonc.2026.1930401

**Published:** 2026-09-30

**Authors:** Ling Fan, Shuang Wu, Xingxing Xie, Yang Zheng, Longxia Chen, Ruilin Ding

**Affiliations:** 1 Departments of Good Clinical Practice, Yaan People’s Hospital, Yaan, Sichuan, China; 2 Department of Anesthesiology, Southwest Medical University, Luzhou, Sichuan, China; 3 Departments of Pharmacy, Yaan People’s Hospital, Yaan, Sichuan, China; 4 Department of Clinical Research Management, Affiliated Hospital of Southwest Medical University, Luzhou, Sichuan, China

**Keywords:** clinical trial, endocrine therapy, immunotherapy, landscape analysis, metastatic castration−resistant prostate cancer, radiopharmaceutical therapy

## Abstract

**Background:**

Metastatic castration-resistant prostate cancer (mCRPC) remains a lethal stage of prostate cancer with poor prognosis. Clinical trials for mCRPC are growing rapidly worldwide. However, a comprehensive systematic analysis of the global research and development landscape in mCRPC remains lacking.

**Methods:**

This study queried the Trialtrove database for interventional trials in mCRPC registered between January 1, 2020 and March 27, 2026. After exclusion of unrelated trials and non-therapeutic studies, 474 trials were analyzed.

**Results:**

The therapeutic landscape for mCRPC has evolved into a highly diversified and dynamic field, overwhelmingly dominated by early-phase investigations. Targeted therapy, particularly PARP inhibitors, remain the leading class, while radiopharmaceutical therapy (RPT), centered on ^177^Lu-PSMA, has reached a mature stage of development. Immunotherapy is expanding beyond checkpoint blockade into bispecific T-cell engagers and cellular therapies, and endocrine therapy is being reshaped by PROTAC degraders. Antibody-drug conjugates (ADCs) are also gaining momentum, with B7 homolog 3 protein (B7-H3) as the most actively pursued targets. Combination regimens account for 39.2% of trials.

**Conclusion:**

mCRPC clinical research landscape has diversified remarkably, shifting from conventional chemotherapy and endocrine therapy toward mechanism-based, biomarker-guided, and combination-oriented strategies.

## 1 Introduction

Prostate cancer continues to exhibit rising trends in both incidence and mortality (1, 2). According to the American Cancer Society, approximately 313,780 new cases of prostate cancer are expected in 2025, along with an estimated 35,770 deaths from the disease (3). Globally, about 10 million men are currently living with prostate cancer, with nearly 700,000 of them having metastatic disease (4).

Although advanced prostate cancer initially depends heavily on androgens and often responds well to androgen deprivation therapy (ADT), most patients eventually progress to metastatic castration-resistant disease (mCRPC), characterized by sustained androgen receptor (AR) activity and rising prostate-specific antigen (PSA) and/or radiographic progression of preexisting disease despite castrate levels of testosterone, leading to poor prognosis and severe declines in quality of life (5–7). Until the early 2010s, docetaxel was the only life-prolonging treatment for mCRPC (8); in the years since, the management of mCRPC has evolved rapidly.

With the results of the PROfound trial (9) published in 2020, the treatment of mCRPC has entered a new era. Currently, the treatment options for mCRPC include novel hormonal agent (NHA) therapy, Antibody-drug conjugate (ADC), targeted therapy, immunotherapy, radiopharmaceutical therapy and chemotherapy (10). Reflecting this expanded landscape, clinical trials for mCRPC are growing rapidly worldwide. However, a comprehensive systematic analysis of the global research and development landscape in mCRPC remains lacking. To fill this gap, we systematically integrated data from major clinical trial registries worldwide and mapped the landscape of mCRPC trials between January 2020 and March 2026.

## 2 Methods

This study systematically analyzed clinical trials in mCRPC from January 1, 2020, using the Trialtrove database (https://clinicalintelligence.citeline.com/), which aggregates trial data from major registries such as ClinicalTrials.gov, ChiCTR, WHO ICTRP, JAPIC and EudraCT. A comprehensive screening of clinical trials registered as of March 27, 2026, was conducted using the following query: (Actual Start Date is from 2020/01/01 to 2026/03/27) OR (Anticipated Start Date is from 2020/01/01 to 2026/03/27)] AND (Study Design does not contain Observation) AND (Patient Segment is Prostate: Hormone refractory). A total of 646 potential studies were initially identified. We excluded trials unrelated to the therapeutic scope of this study, duplicate registrations, and studies evaluating external beam radiotherapy or surgical techniques without a pharmacological intervention. When registry entries contained incomplete or missing information, we supplemented the data by consulting the original trial records or reaching out to the study sponsors. To ensure data integrity and reproducibility, two investigators independently screened and cross-checked the extracted records, and manually harmonized unstructured entries. All conflicts were reconciled through discussion until consensus was reached.

## 3 Results

### 3.1 Overview of the clinical trial landscape in mCRPC

A total of 555 clinical trials were initially identified through the search strategy. Following the exclusion of trials not relevant to the topic and the removal of duplicate records, a total of 474 clinical trials were included in the analysis (**Figure 1**).

**Figure 1:**
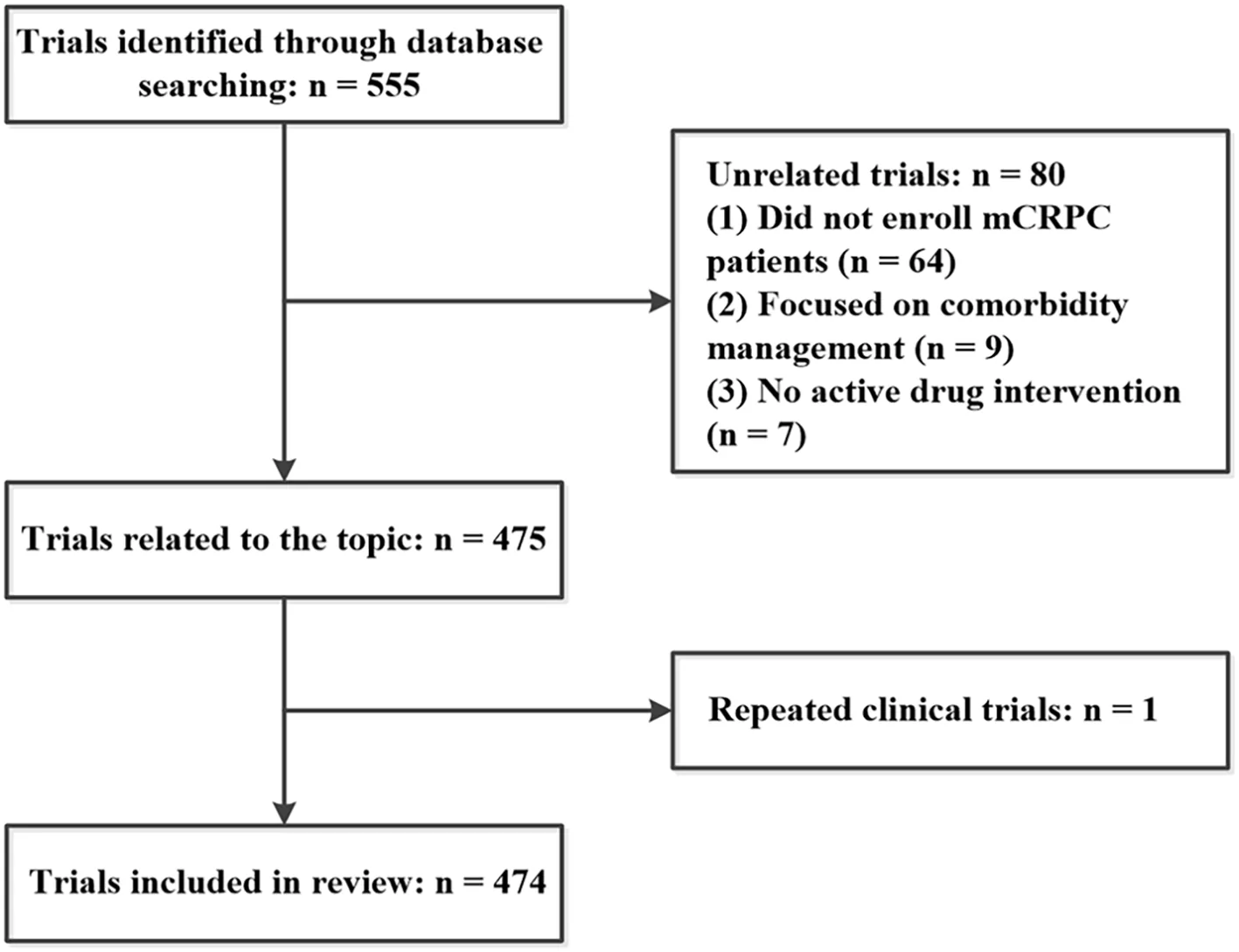
The inclusion and exclusion flowchart.

The temporal analysis reveals a stable trend in mCRPC clinical trials from 2021 to 2025, with approximately 85–95 trials conducted annually (**Figure 2A**). The observed decline in 2026 should be interpreted with caution, as the data collection cutoff date was March 27, 2026. In terms of trial phases (**Figure 2B**), Phase I trials constitute the largest proportion with 183 trials (38.61%), followed by Phase II with 138 trials (29.11%), Phase I/II with 107 trials (22.57%), and Phase III with only 37 trials (7.81%). This phase distribution suggests that the majority of clinical development focuses on dose optimization and preliminary efficacy assessment, with fewer trials advancing to late-stage confirmatory studies. In terms of trial status, active patient recruitment was underway in 239 trials (50.42%), while 57 (12.03%) remained in the preparatory stage. Completed studies accounted for 72 (15.19%), and 51 (10.76%) were classified as closed, including those temporarily suspended. The remaining 55 trials (11.60%) were prematurely discontinued for a variety of reasons (**Figure 2C**). The trial status profile reflects an early-stage yet rapidly evolving research landscape, in which ongoing and preparatory investigations prevail. The majority of the trials (341, 71.94%) were conducted within a single country (**Figure 2D**). As clinical research becomes increasingly globalized, multinational collaborative trials are poised to play a more prominent role. Multinational trials (n = 125) represented 26.37% in this study, with the largest and most diverse study (NCT06136650) spanning 39 countries. Geographically, the United States (n = 253) and China (n = 154) remain the dominant countries for mCRPC clinical research (**Figure 2E**).

**Figure 2:**
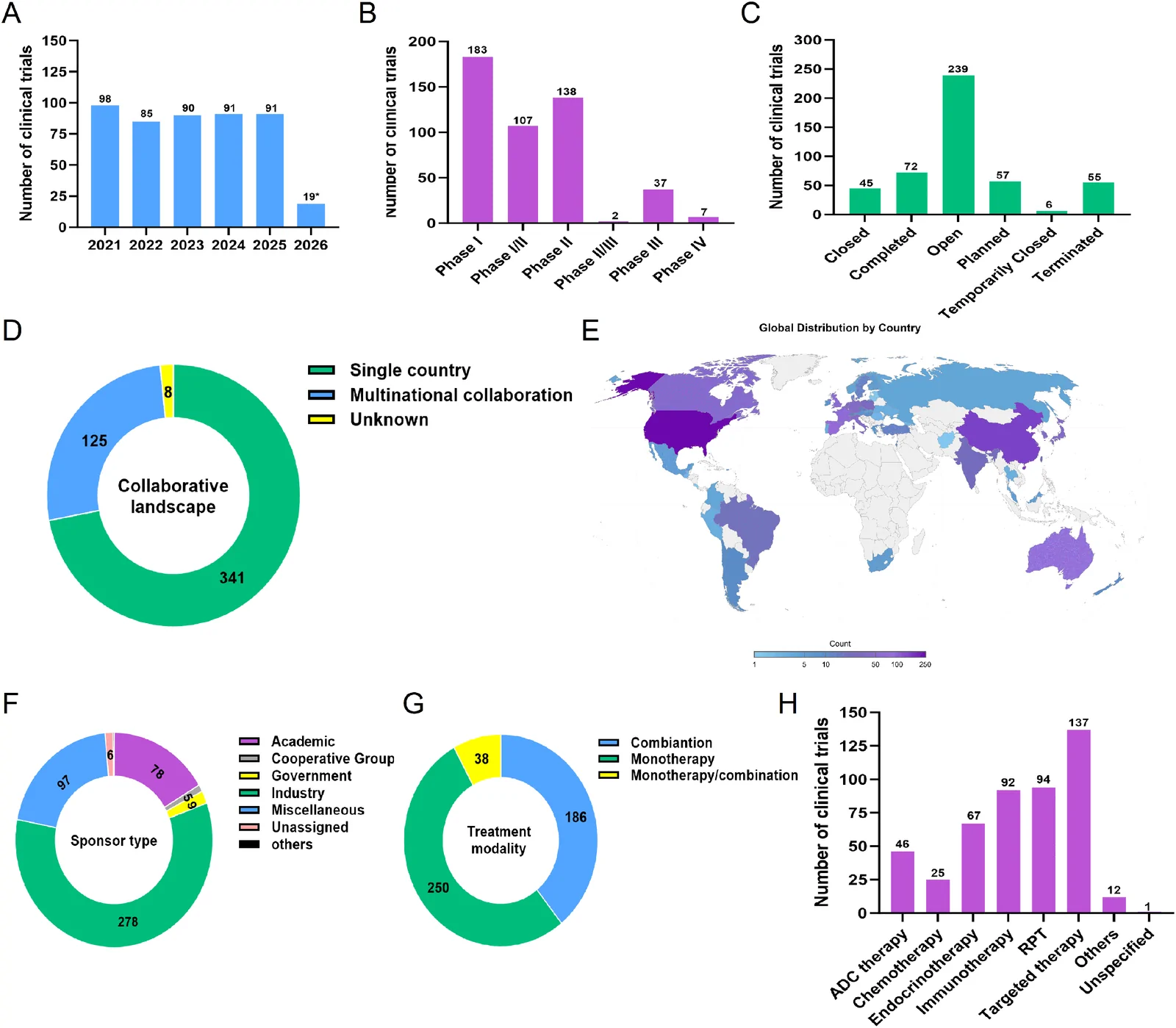
Comprehensive analysis of clinical trials for mCRPC. **(A)** Temporal trends of clinical trials by start years; **(B)** Distribution of clinical trials by clinical stage; **(C)** Distribution of clinical trials by trial status; **(D)** Collaborative landscape of clinical trials; **(E)** Country distribution of clinical trials; **(F)** Sponsor type distribution of clinical trials; **(G)** Treatment modality distribution of clinical trials; **(H)** Drug types of the primary tested agents.

With regard to sponsor type, industry sponsored 58.65% (278/474) of trials (**Figure 2F**). Analysis of treatment modalities revealed that monotherapy regimens were tested in 52.74% (250/474) of trials, while combination regimens were tested in 39.24% (186/474) (**Figure 2G**). In terms of the primary tested drug class (**Figure 2H**), targeted therapy led with 137 trials (28.90%), followed by radiopharmaceutical therapy (PRT, 94, 19.83%), immunotherapy (92, 19.41%), endocrine therapy (67, 14.14%), ADC-based therapy (46, 9.70%), and chemotherapy (25, 5.27%). The remaining 13 trials (2.74%) involved other or unspecified therapeutic types.

### 3.2 Landscape of targeted therapy in mCRPC

The targeted therapy landscape for mCRPC is overwhelmingly dominated by small-molecule agents, which account for 91.97% of trials (126/137; **Figure 3A**). The 137 trials in our analysis explored over 50 distinct targets, 14 of which were assessed in at least two trials (**Figure 3B**). Poly (ADP-ribose) polymerase (PARP) inhibitors emerge as the most prominent class within DNA damage repair targeting, with PARP1/2 dual inhibitors accounting for 36 trials, PARP1-selective inhibitors for 11 trials, and PARP1/2/3 inhibitors for 3 trials (**Figure 3B**). Multi-targeted tyrosine kinase inhibitors (TKIs), designed for the inhibition of multiple oncogenic kinases (e.g., VEGFR, PDGFR, c-Met), account for 15 trials. Additional targets under investigation for mCRPC include bromodomain and extra-terminal (BET) proteins, Cyclin-dependent kinase (CDK)4/6, enhancer of zeste homolog 2 (EZH2), CDK7, AKT, and ataxia-telangiectasia and Rad3-related protein (ATR), each represented by 1–5 trials, indicating exploratory-stage investigation (**Figure 3B**).

**Figure 3:**
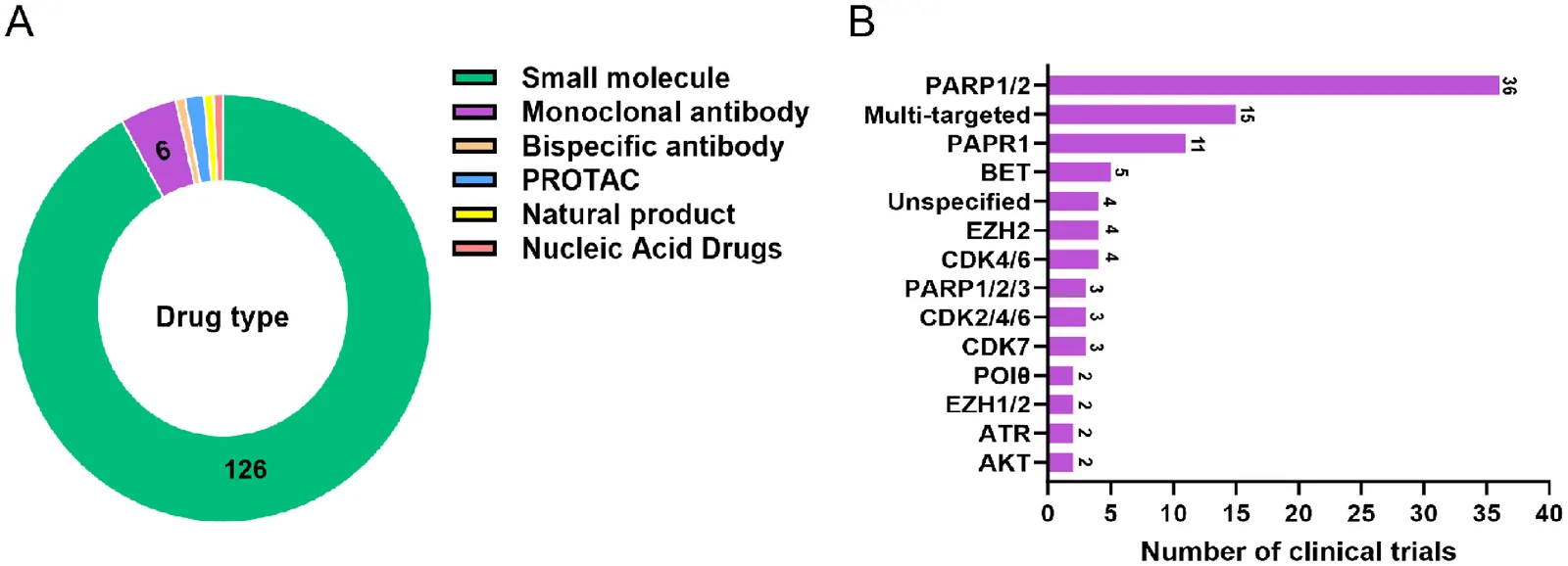
Landscape of targeted therapy in mCRPC. **(A)** Drug type distribution of clinical trials; **(B)** Molecular targets evaluated in at least two interventional trials for mCRPC.

### 3.3 Landscape of radiopharmaceutical therapy in mCRPC

The analysis of the RPT landscape for mCRPC demonstrates a high concentration of registered clinical trial activity centered on prostate-specific membrane antigen (PSMA)-targeted ^177^Lu-based therapies. The nuclide analysis reveals a pronounced dominance of ^177^Lu, which leads all isotopes with 56 (59.57%) clinical trials, accounting for the overwhelming majority of radiopharmaceutical development in mCRPC (**Figure 4A**). ^225^Ac represents the second most investigated nuclide with 18 (19.15%) trials, followed by ^223^Ra, ^212^Pb, ^161^Tb and ^211^At (**Figure 4A**). Target analysis reveals an extraordinary concentration on PSMA, commanding 79 (84.04%) clinical trials (**Figure 4B**). Other targets under investigation include gastrin-releasing peptide receptor (GRPR), B7 homolog 3 protein (B7-H3), kallikrein-related peptidase 3 (KLK3), and Six-transmembrane epithelial antigen of the prostate-2 (STEAP2), each represented by 1–3 trials, indicating early-stage exploration of these alternative targets (**Figure 4B**). The correlation between targets and nuclides is shown in **Figure 4C**. Beta-emitter radioisotopes accounted for the majority of clinical trials in this field (63 trials, 67.02%, **Figure 4C**), underscoring the continued dominance of beta-emitting nuclides, particularly ¹^77^Lu, in the current RPT landscape for mCRPC.

**Figure 4:**
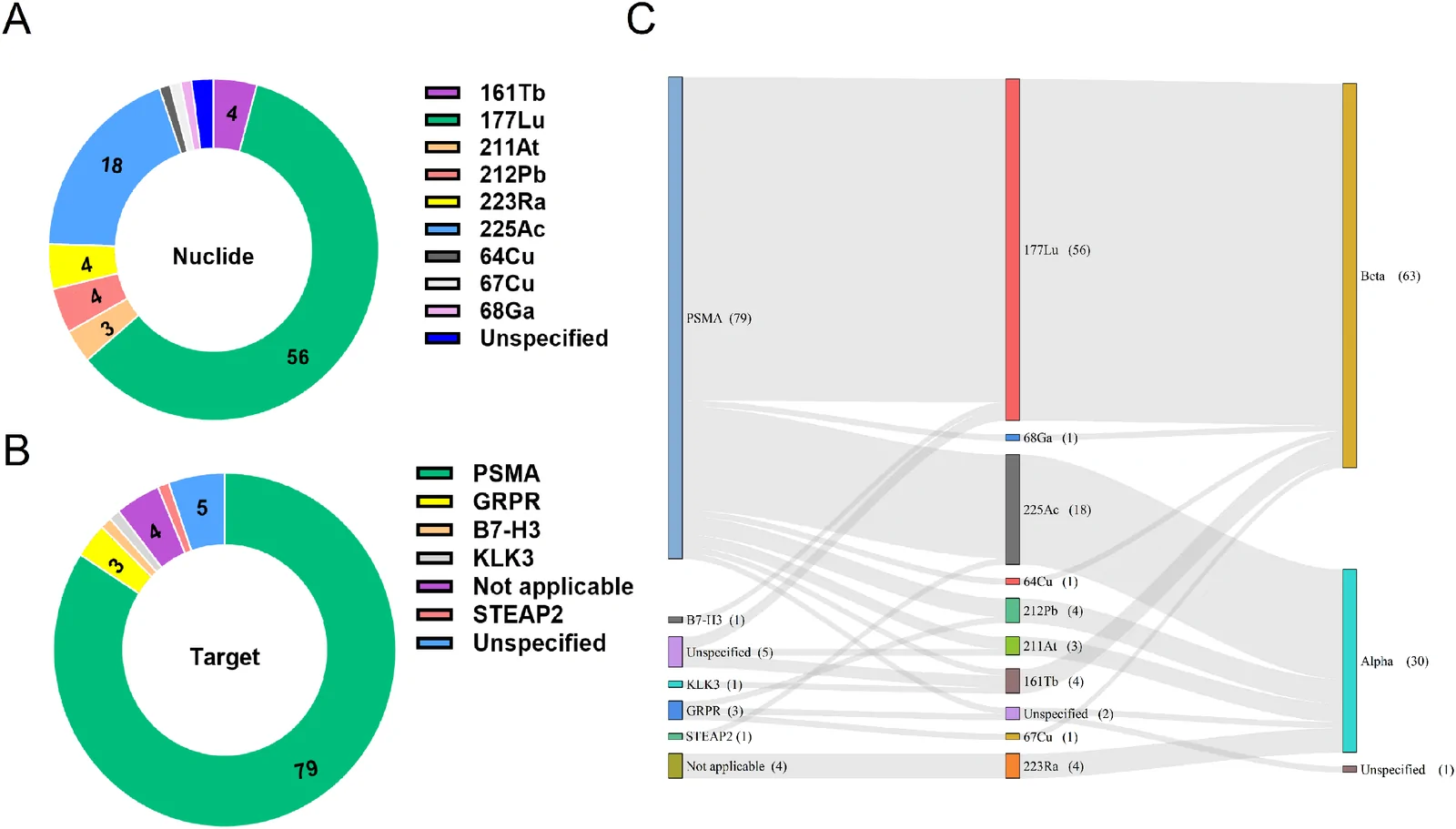
Landscape of radiopharmaceutical therapy in mCRPC. **(A)** Distribution of nuclides evaluated in radiopharmaceutical therapy trials for mCRPC; **(B)** Molecular targets pursued in radiopharmaceutical therapy trials for mCRPC; **(C)** The relationship between targets and nuclides in radiopharmaceutical therapy trials.

### 3.4 Landscape of immunotherapy in mCRPC

The immunotherapy landscape for mCRPC shows remarkable diversity in therapeutic modalities. Bispecific antibodies lead with 26 trials, followed by cell therapy (24 trials), monotherapy (18 trials), anticancer vaccines (12 trials), small molecules (9 trials), trispecific antibodies (2 trials), recombinant interleukin (2 trials), recombinant interferon (1 trial), and oncolytic virus (1 trial) (**Figure 5A**). Cell therapy accounts for 24 trials, predominantly directed against PSMA (**Figure 5B**). Other prostate antigens, such as prostatic acid phosphatase (PAP) and prostate stem cell antigen (PSCA), are also promising targets for cell therapy in mCRPC. Bispecific antibodies, meanwhile, exhibit diverse target combinations, including PSMA/CD3, KLK2/CD3, PD-1/CTLA-4, PSMA/CD28, and B7-H3/CD28, reflecting the breadth of antigen-targeting approaches (**Figure 5C**). Monoclonal antibodies comprise 18 trials, mainly targeting established immune checkpoint molecules such as programmed death 1 (PD-1, 12 trials) and cytotoxic T-lymphocyte antigen-4 (CTLA-4, 2 trials) (**Figure 5D**). Other immune regulatory molecules under investigation as targets for monoclonal antibodies in mCRPC include butyrophilin 3A1 (BTN3A1), leukocyte immunoglobulin-like receptor subfamily B member 2 (LILRB2) and ENTPD1 (CD39) (**Figure 5D**). Trispecific antibodies, by contrast, remain an emerging modality with only 2 trials initiated to date, underscoring the early stage of this approach within the mCRPC immunotherapy landscape (**Figure 5E**).

**Figure 5:**
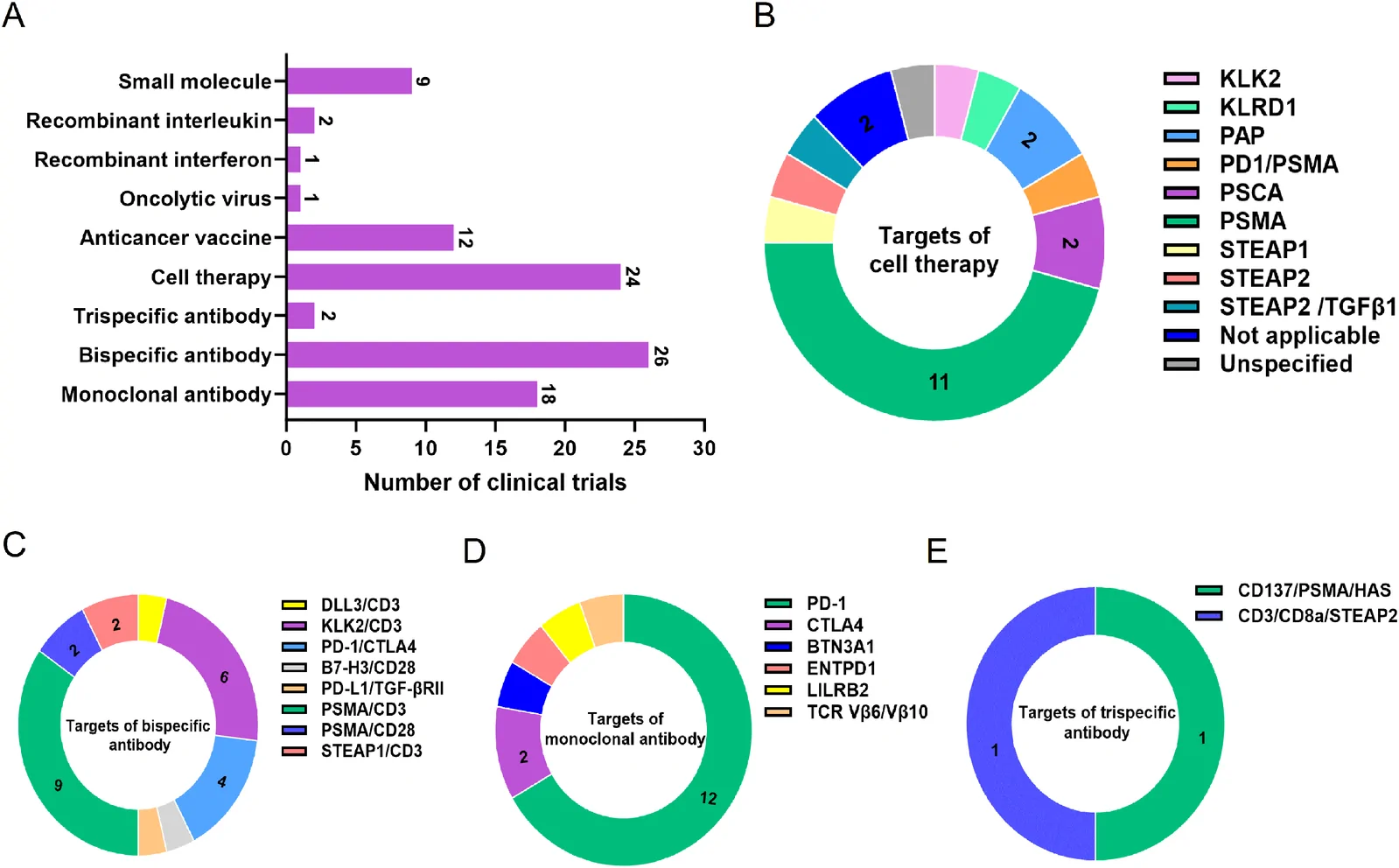
Landscape of immunotherapy in mCRPC. **(A)** Distribution of immunotherapy modalities in mCRPC clinical trials; **(B)** Targets of cell-based immunotherapies in mCRPC trials; **(C)** Targets of bispecific antibody-based immunotherapies in mCRPC; **(D)** Targets of monoclonal antibody-based immunotherapies in mCRPC; **(E)** Targets of Trispecific antibody-based immunotherapies in mCRPC.

### 3.5 Landscape of endocrine therapy in mCRPC

The endocrine therapy landscape is centered on the AR pathway, with 38 trials (56.72%) directed at AR-related mechanisms. Among AR-targeted agents, small molecules account for 23 trials, comprising traditional AR antagonists and inhibitors (**Figure 6**). Notably, PROTAC (Proteolysis-Targeting Chimera) technology accounts for 15 trials, making up 39.47% of the AR-targeted trials. CYP17A1 inhibition accounts for 14 trials, all of which employ small molecule inhibitors, while CYP11A1 targeting accounts for 8 trials (**Figure 6**).

**Figure 6:**
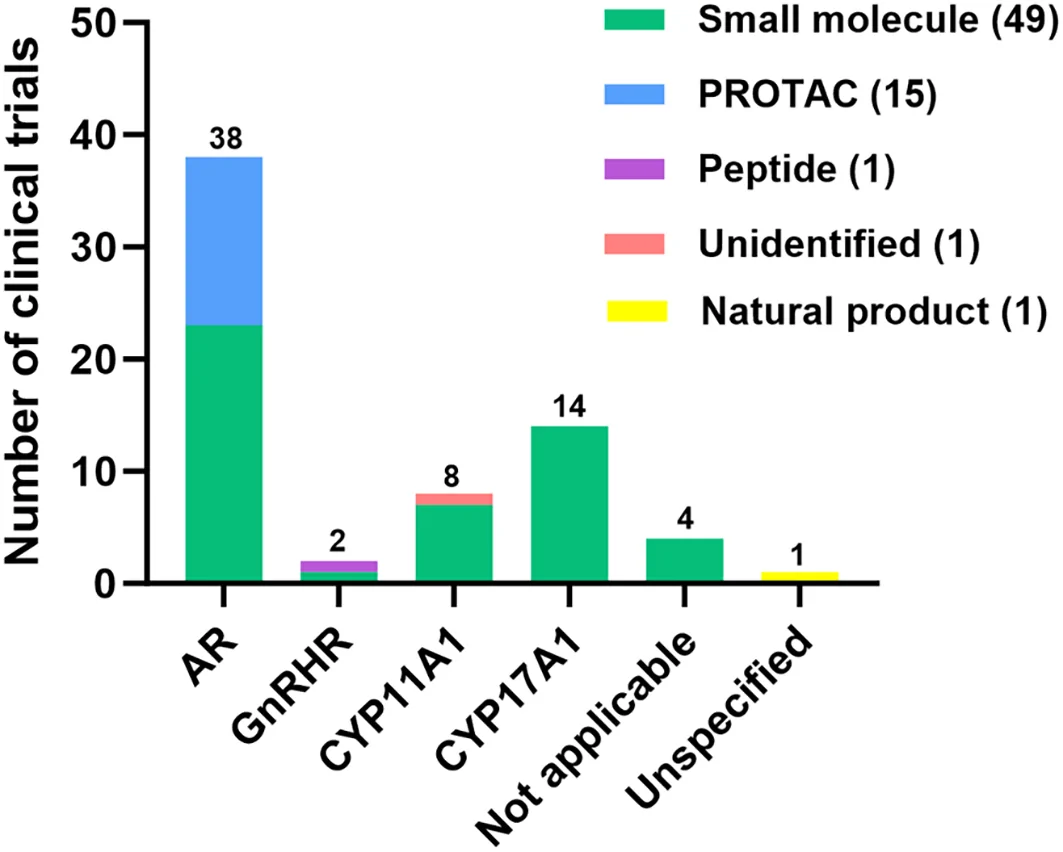
Targets and drug classes of endocrine therapy trials in mCRPC.

### 3.6 Landscape of ADC drug development in mCRPC

The ADC pipeline in mCRPC shows clear target prioritization. B7-H3 is the most actively pursued target, accounting for 13 trials (28.26%), followed by HER2-targeting ADCs with 6 trials (13.04%). Trophoblast cell surface antigen 2 (TROP2) and prostate-PSMA-targeting ADCs each account for 4 trials (8.70%). Other targets under investigation include HER3, six-transmembrane epithelial antigen of the prostate 1 (STEAP1), TAG-72, NECTIN4, EGFR/HER3, CD46, 5T4, and ADAM9 (**Figure 7**).

**Figure 7:**
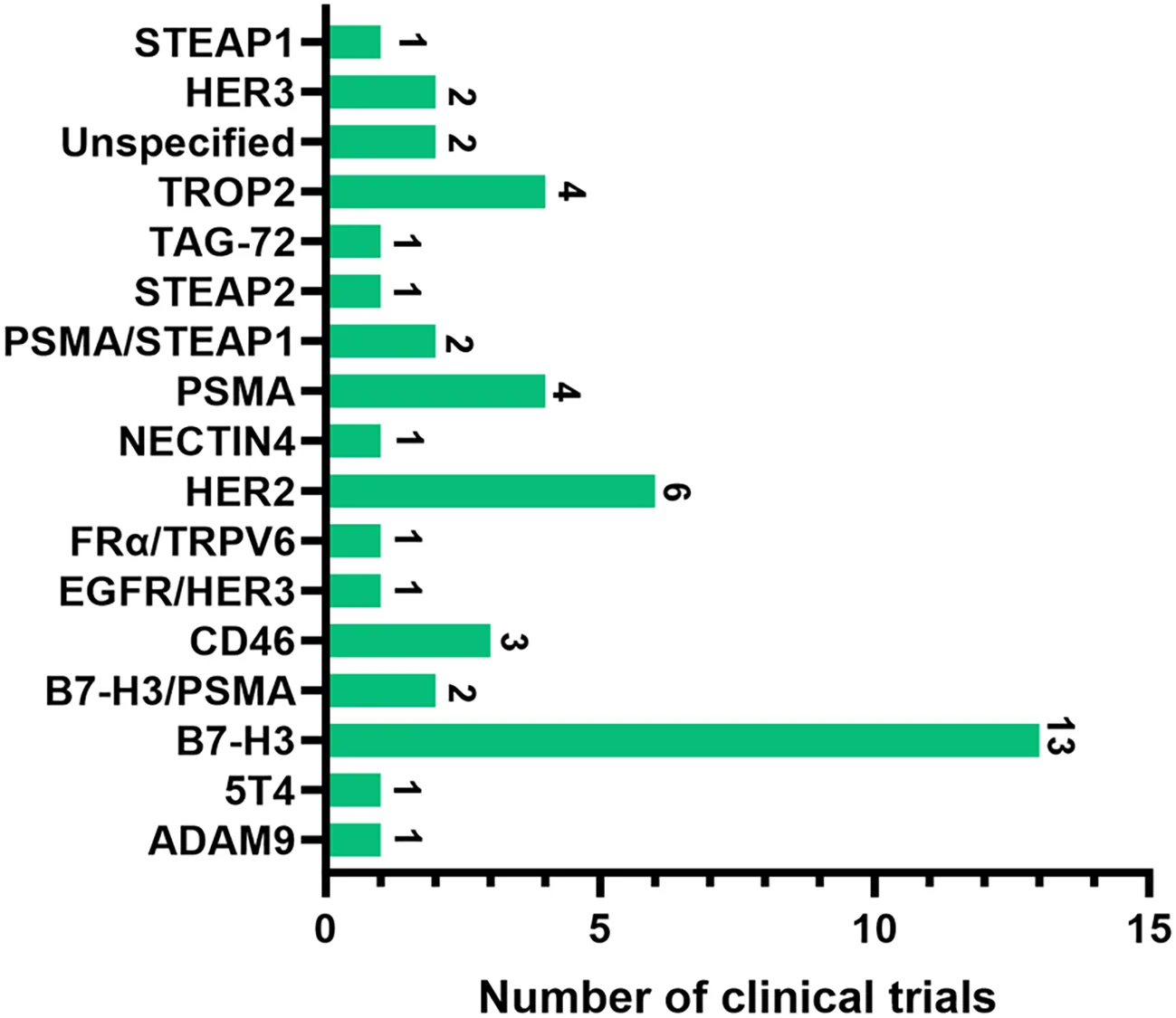
Targets of antibody–drug conjugate trials in mCRPC.

## 4 Discussion

mCRPC remains a challenging disease to treat, associated with poor patient outcomes (11). In this study, we systematically analyze the current therapeutic landscape for mCRPC. The results reveal that mCRPC clinical development is vibrant and multifaceted, moving far beyond the classic reliance on ADT and taxane-based chemotherapy. While much of the pipelines remain in early investigation, the convergence of RPT, PROTAC, cell therapy, bispecific immune engagers, and novel ADCs promises a new generation of treatment options that will further personalize and potentiate care for men with mCRPC.

The temporal analysis reveals a stable trend in mCRPC clinical trials from 2021 to 2025, with approximately 85–95 trials conducted annually. The apparent decline in 2026 is due to the cutoff date of March 27, 2026, which means only part of the year’s trials are captured. This is a known limitation often seen in ongoing trial landscape analyses. The phase distribution analysis reveals a striking predominance of early-phase clinical trials, with Phase I accounting for 38.61% of all enrolled trials. Phase III trials constitute merely 7.81%. This phase distribution suggests that the current mCRPC pipeline remains largely concentrated in early exploratory stages, with dose optimization and preliminary efficacy assessment dominating the clinical development landscape. The large proportion of trials actively recruiting (50.42%) or in planning (12.03%) further supports the characterization of mCRPC clinical research as nascent yet rapidly advancing. Notably, the 11.60% trial termination rate observed in our analysis aligns with industry benchmarks for oncology clinical development, where approximately 8-30% of initiated trials are discontinued due to factors including insufficient efficacy, safety concerns, strategic business decisions, or enrollment challenges (12–14). The high rate of single-country trials suggests that many studies are designed for regional regulatory purposes or as initial first-in-human explorations; nevertheless, the presence of 125 multinational trials, aligns with the growing emphasis on global collaborative research to expedite accrual and enhance generalizability (15). Industry sponsorship for 58.65% of trials indicates robust commercial investment, which is essential for translating early academic findings into late-stage development and eventual clinical application. The therapeutic class analysis demonstrates that targeted therapy leads with 28.90% of trials, followed by RPT, immunotherapy, endocrine therapy, ADC therapy and chemotherapy.

The prominence of targeted agents is largely driven by small molecules, particularly PARP inhibitors. Preclinical studies have revealed reciprocal crosstalk between the AR pathway and the DNA damage response (DDR). AR blockade downregulates the expression of homologous recombination repair (HRR) genes, shifting cellular dependence toward PARP-mediated repair; conversely, PARP inhibition can attenuate AR signaling (16). This biological foundation has provided the impetus for evaluating several PARP inhibitors in the mCRPC setting. Phase III trials, such as PROpel (NCT03732820) (17), TALAPRO-2 (NCT03395197) (18), and MAGNITUDE (NCT03748641) (19), have demonstrated clinically meaningful benefit, particularly in tumors harboring BRCA1/2 alterations. Our analysis showed that PARP1/2 inhibitors accounted for 36 trials, consisting of both novel drug investigations and generic bioequivalence studies. In contrast, PARP1-selective and PARP1/2/3 inhibitors represented a smaller, more recently emerged set of investigational agents. Evidence indicates that selective PARP1 trapping, rather than dual PARP1/2 inhibition, is sufficient to trigger synthetic lethality in HRD-positive tumor cells (20). Consequently, next-generation PARP1-selective inhibitors such as saruparib achieve enhanced antitumor activity while mitigating the PARP2-associated hematologic toxicity seen with earlier pan-PARP inhibitors (21, 22). From a biological standpoint, the benefit of PARP inhibition is most pronounced in tumors with BRCA1/2-driven homologous recombination deficiency (HRD), in which loss of BRCA-mediated repair creates a synthetic-lethal dependency on PARP activity (16, 20). Accordingly, patients harboring BRCA1/2 alterations constitute the molecular subgroup most likely to derive meaningful benefit, and contemporary trial design increasingly mandates prospective HRR genotyping. By contrast, responses in tumors harboring non-BRCA alterations, such as ATM or CDK12, have been limited and heterogeneous across studies (19), underscoring that HRR gene status alone is an imperfect surrogate for HRD and that functional or genomic-scar-based biomarkers are needed to further refine patient selection.

Beyond PARP, over 50 additional targets are currently under investigation for the targeted therapy of mCRPC. Among them, the PI3K/AKT pathway has drawn considerable attention. It has been reported that PTEN loss occurs in 40-60% of patients with mCRPC, causing activation of the PI3K/AKT signaling pathway (6). Dual inhibition of PI3K/AKT and AR pathways represents another rational combination strategy aimed at overcoming adaptive resistance in mCRPC. The AKT inhibitor ipatasertib has shown modest activity in combination with abiraterone in PTEN-loss mCRPC, and further optimization is ongoing (23). Another key target is CDK, which enhances AR transcriptional activity (24). Ongoing trials in mCRPC aim to refine CDK4/6 inhibitor strategies, develop new inhibitors of CDK2 and CDK7, and explore combinations with other targeted pathways (25). Similarly, the epigenetic regulator EZH2 potentiates AR signaling through an atypical, non-enzymatic mechanism and governs lineage plasticity, such that its inhibition restores responsiveness to AR-targeted agents (26). EZH2 inhibitors such as mevrometostat are under clinical investigation in combination with AR inhibitors, with phase III trials already ongoing (NCT06551324 and NCT06629779) (27).

In our analysis, the RPT landscape is characterized by a marked concentration of registered trial activity on PSMA-targeted ^177^Lu-based therapies. This predominance reflects the concentration of registered research activity and, by itself, does not establish clinical maturity; the clinical interpretation that follows is drawn from the published literature rather than from the registry analysis itself. ^177^Lu-PSMA therapy has been shown to reduce PSA levels and improve overall survival (OS) in patients with mCRPC. For instance, the phase III VISION trial (NCT03511664) demonstrated that the addition of ^177^Lu-PSMA-617 significantly prolonged OS and radiographic progression-free survival (PFS) in heavily pretreated mCRPC patients, ultimately leading to global regulatory approvals of ¹^77^Lu-PSMA-617 (Pluvicto™) (28). In the phase III PSMAfore trial (NCT04689828), ¹^77^Lu-PSMA-617 was associated with a significant prolongation of radiographic PFS versus AR inhibitors, alongside a manageable safety profile (29). The beta-emitting radioisotope ^177^Lu exhibits a mean energy of 133.6 keV, a maximum tissue penetration depth of less than 2 mm, and an approximate half-life of 6.64 days, which falls within the optimal range for therapeutic applications (8, 30). Its favorable physicochemical profile, combined with reliable production and supply, positions it as an emerging theranostic radionuclide of choice (31). Our analysis shows that ¹^77^Lu accounts for 59.57% of all RPT trials during the study period. Although ^177^Lu remains the dominant beta emitter in RPT agent development, ^161^Tb is emerging as a compelling alternative owing to its comparable beta energy and half-life (32). Beyond beta emission, ^161^Tb also releases Auger and internal conversion electrons, which are thought to enhance DNA damage (33). ^161^Tb-PSMA-617 has shown greater therapeutic efficacy than ^177^Lu-PSMA-617 in preclinical models, both *in vitro* and *in vivo (*34); whether this preclinical advantage translates into superior clinical outcomes has not been established and requires prospective clinical evaluation. As expected, 4 trials employing ^161^Tb-based RPT were registered during the study period. In contrast to beta emitters, alpha particles deliver highly localized cytotoxic doses owing to their high linear energy transfer and short penetration depth (50-100 µm) (35). This property has led to the investigation of alpha emitters as a potential option for patients whose disease has become refractory to beta-radiation therapy (33); however, such a treatment-sequencing strategy has not yet been established clinically and requires prospective validation. Alpha-emitting radionuclides currently being evaluated in clinical trials for mCRPC include ^225^Ac, ^212^Pb, and ^211^At. Our analysis identified 18 trials involving ^225^Ac-based therapy, accounting for 19.15% of all RPT trials. This positions ^225^Ac as the most frequently investigated alpha emitter in the mCRPC landscape. Data from a phase I trial demonstrated safety and preliminary efficacy signals of ^225^Ac-based RPT in progressive mCRPC (36).

An optimal theranostic target should be highly expressed on prostate cancer cells with minimal normal tissue distribution, and possess a ligand that can be labeled with radionuclides (37). In addition, stable binding and efficient ligand-receptor internalization further enhance the cytotoxic effect of RPT (37). Currently, PSMA meet nearly all of these criteria. In our analysis, PSMA, a 750-amino acid transmembrane protein, is identified as the most extensively studied target for RPT in mCRPC, accounting for 79 trials (84.04% of all RPT studies). It has been reported that PSMA is overexpressed 100- to 1000-fold in prostate cancer compared with normal prostate tissue, and its expression is further upregulated in mCRPC, making it an ideal theranostic target for RPT (8, 38). However, PSMA is not strictly prostate-specific. It is also expressed in normal tissues, including the salivary glands, kidneys, and small intestine, posing a risk of off-target radiation exposure (39). Furthermore, several biological and clinical limitations of PSMA-targeted RPT warrant emphasis. First, PSMA expression is highly heterogeneous both between patients and among lesions within the same patient, and a clinically relevant proportion of mCRPC cases exhibit PSMA-negative or PSMA-low disease that is intrinsically unsuitable for PSMA-directed therapy (40–42). Second, treatment-emergent neuroendocrine differentiation, frequently accompanied by loss of PSMA expression and AR signaling, represents an increasingly recognized mechanism of resistance and a distinct clinical state requiring alternative therapeutic strategies (6). Third, acquired resistance to ^177^Lu-PSMA-617 may also arise through impaired DNA damage repair capacity, target downregulation, and clonal selection of PSMA-negative cells, while the optimal sequencing of RPT with AR pathway inhibitors, chemotherapy, and PARP inhibitors remains undefined (8, 30). These considerations highlight the need for biomarker-guided patient selection, rational combination and sequencing strategies, and the continued development of non-PSMA targets. Currently, several alternative antigens are under investigation, including GRPR, B7-H3, KLK3, and STEAP2 (43).

The endocrine therapy landscape remains firmly anchored to the AR pathway, with 56.72% of trials directly targeting AR-related mechanisms. While next-generation nonsteroidal AR signaling inhibitors, such as enzalutamide, apalutamide and darolutamide, are well-established (44–46), our analysis highlights the emergence of PROTACs as a significant therapeutic advancement in mCRPC. In our analysis, 39.47% of the AR-directed trials involve PROTACs, which are bifunctional chimeric degraders featuring one domain for the protein of interest and another for E3 ubiquitin ligase (47, 48). PROTACs eliminate target proteins independently of an active binding pocket, enabling durable target suppression with a prolonged duration of action and a broader therapeutic index (47). Conventional AR antagonists frequently give rise to resistance, mediated by mechanisms such as AR gene amplification, ligand-binding domain (LBD) mutations, and the emergence of constitutively active AR splice variants (AR-Vs) (47, 49, 50). PROTACs can simultaneously degrade both full-length AR and the AR-Vs, largely bypassing the constraints imposed by single-site mutations. This property endows PROTACs with unique potential in overcoming acquired resistance driven by AR pathway alterations (47). ARV-110 is the first AR-targeting PROTAC to enter clinical trials for mCRPC. In a phase I/II trial (NCT03888612), it demonstrated promising clinical activity in heavily pretreated patients with acquired resistance to conventional AR inhibitors (51, 52). ARV-766, a second-generation PROTAC, exhibits improved stereochemical stability and superior degradation potency against a broad panel of clinically relevant AR mutants compared with ARV-110. This orally bioavailable agent entered a phase I/II clinical trial in 2021 to evaluate its safety and efficacy (NCT05067140) (53). Other AR-targeting PROTACs that have advanced to clinical trials for mCRPC include CC-94676, HP518, and HSK38008.

Beyond bioavailability, several unresolved issues currently limit the translational maturity of AR-directed PROTACs. Although degradation of full-length AR has been demonstrated clinically, activity against ligand-binding-domain-truncated AR splice variants such as AR-V7, which lack the canonical ligand-binding domain targeted by many current degrader designs, remains uncertain and may require alternative warhead or E3-ligase recruitment strategies (47–49). Pharmacokinetic optimization is likewise incomplete: the high molecular weight and poor drug-like properties of PROTACs constrain oral exposure and tissue penetration, and dose-limiting toxicities have occasionally narrowed the achievable therapeutic window (47, 48). Finally, acquired resistance mechanisms, including alterations in E3 ubiquitin ligase complexes and restoration of AR signaling through parallel pathways, are only beginning to be characterized and will likely shape the design of next-generation degraders and combination regimens (49, 50).

Additional interest in androgen biosynthesis inhibition continues, with CYP17A1 and CYP11A1 inhibitors being investigated, although the frontline adoption of abiraterone (a selective inhibitor of CYP17A1) (54) has made the clinical development path for new steroidogenesis inhibitors more challenging. CYP17A1, a key enzyme in androgen synthesis, has long been a validated therapeutic target in prostate cancer. More recently, attention has shifted to CYP11A1, an upstream enzyme that catalyzes the conversion of cholesterol to pregnenolone, the initial and rate-limiting step in steroidogenesis (55, 56). Our analysis identified 8 trials of CYP11A1 inhibitors, reflecting emerging interest in this target. Opevesostat (MK-5684, formerly ODM-208) is a first-in-class, oral CYP11A1 inhibitor. The phase I/II CYPIDES trial (NCT03436485) evaluated opevesostat in heavily pretreated patients with mCRPC and demonstrated preliminary antitumor activity, particularly in those harboring activating AR LBD mutations (57). Based on these results, the open-label phase III trials (NCT06136624 and NCT06136650) were initiated to evaluate opevesostat versus alternative AR inhibitors in mCRPC patients (58).

Although immune checkpoint inhibitors (ICIs) have demonstrated limited single-agent efficacy in unselected mCRPC populations (59, 60), our data reveal a striking diversification of immunotherapeutic modalities. This shift reflects the need to overcome the immunologically “cold” tumor microenvironment of prostate cancer (59), which has driven exploration beyond conventional checkpoint blockade into bispecific T-cell engagers (TCEs), cellular therapies, cancer vaccines, and other innovative platforms. In our analysis, monoclonal antibodies against established ICIs continue to be extensively evaluated in mCRPC. However, there is a clear trend toward evaluating ICI-based combination strategies, including combinations with ADT, chemotherapy, RPT, PARP inhibitors, and other treatment modalities (60). Bispecific antibodies lead with 26 trials, primarily targeting PSMA/CD3, followed by KLK2/CD3 and PD-1/CTLA-4. TCEs, most commonly bispecific antibodies, concurrently target CD3 on T cells and a TAA on neoplastic cells, creating an artificial immunological synapse that tethers the two cell types. PSMA/CD3 TCEs, including acapatamab, pasotuxizumab, and JNJ-63898081, induce T-cell activation and restrict cytotoxic activity to prostate cancer cells in a PSMA-dependent manner (61–63). Early-phase trials of PSMA/CD3 TCEs have shown preliminary antitumor activity, with cytokine release syndrome being generally manageable (61–63). However, the durability of responses of PSMA/CD3 TCEs in mCRPC requires further evaluation in larger, longer-term studies. Beyond PSMA/CD3, the exploration of other bispecific constructs, including KLK2/CD3 and B7-H3/CD28, reflects a dual strategy: engaging alternative TAAs while simultaneously providing co-stimulatory signals to counteract T-cell exhaustion. Notably, two trials involving trispecific antibodies were registered during the study period. Trispecific antibodies represent an emerging class of immunotherapeutic agents that redirect adaptive effector cells to tumor cells and enhance anti-tumor immunity (64). CB307 is a trispecific antibody that engages PSMA, CD137, and human serum albumin (HSA), and is being developed for PSMA-positive solid tumors including mCRPC. By conditionally activating T cells only in PSMA-expressing tumors, this design is intended to mitigate hepatotoxicity, while HSA binding extends the drug’s half-life (65). Preliminary results from a phase I trial have demonstrated tumor responses in patients with mCRPC (65). The other trispecific antibody in clinical evaluation for mCRPC is directed against CD3, CD8a, and STEAP2. Despite this progress, the clinical translation of T-cell engagers in mCRPC faces substantive barriers. Antigen heterogeneity and treatment-induced downregulation of target antigens such as PSMA may permit immune escape (41, 61). The immunosuppressive tumor microenvironment of prostate cancer, characterized by sparse T-cell infiltration, abundant suppressive myeloid populations, and TGF-β-mediated immune exclusion, further limits effector function (66, 67). T-cell exhaustion driven by continuous antigen exposure, on-target off-tumor toxicity, and cytokine release syndrome constrain dosing and durability, and response persistence has generally been short in early-phase studies (61–63). These challenges motivate the exploration of conditionally activated constructs, co-stimulatory engagement, and combinations with agents that remodel the tumor microenvironment.

To overcome these barriers, cell therapy, particularly CAR T-cell therapy, has emerged as a promising strategy, focusing on prostate-associated antigens such as PSMA, PAP, STEAP1 and PSCA (66). Our analysis identified 24 cell therapy trials, predominantly targeting PSMA. The early-phase trials of PSMA-targeting CAR-T cells have reported encouraging preliminary efficacy. For instance, a phase I trial (NCT04249947) of P-PSMA-101 CAR-T cells in patients with mCRPC demonstrated ≥ 50% PSA declines in 21% of patients, including two who achieved durable remissions exceeding 12 months with PSA reductions greater than 90% (68). Furthermore, a phase I trial (NCT03089203) evaluating PSMA-targeted, TGFβ-insensitive armored CAR-T cells in mCRPC reported that approximately 30% of treated patients achieved a ≥ 30% PSA decline, including one patient who experienced a rapid and profound PSA reduction exceeding 98% (69). Other prostate antigens for CAR-T therapy such as PAP and PSCA remain viable targets, providing alternative options when PSMA loss or heterogeneity emerges. However, challenges in treating mCRPC with CAR-T cells persist, including limited *in vivo* persistence and severe toxicity, which underscore the need for continued optimization of CAR engineering and safety-switching strategies. In addition, antigen heterogeneity and treatment-induced antigen loss may undermine single-target CAR-T approaches, while the immunosuppressive microenvironment and limited T-cell trafficking to bone-dominant metastatic sites impair effector-cell accumulation and function (66, 67). T-cell exhaustion, cytokine release syndrome, and the logistical burden and cost of autologous cell manufacturing further constrain near-term clinical applicability (68, 69). Collectively, these factors indicate that CAR-T therapy in mCRPC remains a highly experimental modality whose routine clinical implementation is unlikely in the near term.

ADCs are a novel class of biotherapeutics linking the specificity of monoclonal antibodies with potent cytotoxic payloads, thereby enhancing antitumor efficacy while minimizing systemic toxicity (70). Among ADCs under clinical evaluation for mCRPC, B7-H3 (CD276) is the most actively pursued target, accounting for 28.26% of trials. It has been reported that B7-H3 is highly expressed on prostate cancer cells and tumor vasculature, with minimal expression in normal tissues, making it an attractive therapeutic target for mCRPC (70–72). Early-phase trials of B7-H3 ADCs have shown promising objective responses in mCRPC. For instance, ifinatamab deruxtecan, a potential first-in-class B7-H3-targeting ADC, has demonstrated encouraging activity in heavily pretreated mCRPC: in a phase I/II trial (NCT04145622), 15 of 59 evaluable patients achieved a partial or complete response per RECIST (73). DB-1311/BNT324, another B7-H3-targeting ADC, has shown encouraging efficacy and a manageable safety profile in heavily pretreated patients with mCRPC in a phase I/II trial (NCT05914116). Among 58 response-evaluable patients, the confirmed ORR was 34.5% (74).

HER2-targeted ADCs continue to be evaluated, despite the low frequency of HER2 amplification in metastatic prostate cancer (75), possibly due to bystander killing effects and payload-driven efficacy. TROP2 and PSMA-targeting ADCs each represent 8.70% of the trials. Sacituzumab govitecan, a TROP2-targeting ADC, has received FDA approval for urothelial carcinoma, and its activity in mCRPC is currently under investigation (NCT03725761) (76). Datopotamab deruxtecan (Dato-DXd), another Trop-2 ADC with a DXd payload, is being assessed in mCRPC within the phase II TROPION-PanTumor03 trial (NCT05489211) (77). ARX517, a PSMA-targeting ADC, resulted in PSA declines and objective responses in patients with mCRPC enrolling in a first-in-human study (NCT04662580), providing early proof-of-concept for this approach. The growing diversity of targets under investigation, including STEAP1, Nectin-4, CD46, and HER3, further illustrates the expanding applicability of ADC technology to mCRPC. Notably, 6 trials investigating bispecific ADCs were registered during the study period, targeting pairs such as PSMA/STEAP1, B7-H3/PSMA, EGFR/HER3, and FRα/TRPV6. By engaging two distinct antigens or epitopes, bispecific ADCs enhance tumor selectivity and internalization efficiency, positioning them as a promising direction for next-generation ADC development (78).

Combination therapy represents a major and biologically rational theme in the current mCRPC landscape, accounting for 39.2% of trials in our analysis, and several dominant combination patterns can be discerned. First, AR pathway inhibitors plus PARP inhibitors constitute the most clinically advanced combination strategy, grounded in the reciprocal crosstalk between AR signaling and the DNA damage response, and validated by the phase III PROpel, TALAPRO-2, and MAGNITUDE trials, with the greatest benefit observed in HRR-deficient tumors (16–19). Second, radioligand therapy combined with AR-targeted agents is being explored on the premise that AR blockade may modulate PSMA expression and radiosensitize tumor cells, whereas radioligand-induced DNA damage may delay the emergence of AR-driven resistance (28–30). Third, immunotherapy combined with PARP inhibition or radioligand therapy aims to convert the immunologically cold prostate tumor microenvironment into a more inflamed state through increased antigen release and DNA-damage-induced immune activation (59, 60). Fourth, ADC-based combinations, particularly with AR pathway inhibitors or immunotherapy, seek to exploit payload-mediated cytotoxicity alongside complementary mechanisms of action (70). These patterns indicate that combination development in mCRPC is increasingly mechanism-driven rather than empirical, although the optimal sequencing, patient selection, and management of cumulative toxicity remain to be defined.

To move beyond a descriptive catalogue and to provide an integrative, clinically oriented perspective, **Table 1** summarizes the major therapeutic classes according to their biological rationale, key predictive biomarkers, representative agents, current clinical stage, estimated proximity to routine clinical use, and principal barriers to translation.

**Table 1:** Integrative summary of major therapeutic classes in the mCRPC clinical trial landscape.

Therapeutic class	Biological rationale	Key biomarker	Representative agents	Clinical stage*	Proximity to routine clinical use	Major barriers to translation
PARP inhibitors	Synthetic lethality in HRD tumors; reciprocal AR-DDR crosstalk	BRCA1/2 alteration	Olaparib, niraparib, talazoparib; next-generation PARP1-selective agents (e.g., saruparib)	Phase III completed; approved	Already in routine clinical practice	Acquired resistance; imperfect biomarkers for non-BRCA HRD; hematologic toxicity
PSMA-targeted radioligand therapy	Targeted delivery of beta/alpha radiation to PSMA-expressing tumor cells	PSMA expression on PET imaging	177Lu-PSMA-617; 225Ac-PSMA; 161Tb-PSMA	Phase III completed; approved	Already in routine clinical practice; expanding to earlier lines	PSMA heterogeneity; PSMA-negative lesions; treatment-emergent neuroendocrine differentiation; acquired resistance
Next-generation AR pathway inhibitors	Blockade of androgen receptor signaling	AR expression; AR LBD mutations (resistance)	Enzalutamide, apalutamide, darolutamide	Approved	Standard of care	AR amplification; LBD mutations; constitutively active splice variants
AR-directed PROTAC degraders	Ubiquitin-proteasome-mediated degradation of full-length AR	AR LBD mutations; predictive value of AR-V status uncertain	ARV-110, ARV-766, CC-94676, HP518	Phase I/II	Experimental	Oral bioavailability and pharmacokinetics; activity against AR splice variants; E3-ligase-mediated resistance
T-cell engagers (bi-/trispecific antibodies)	Redirection of T cells to tumor antigens via CD3 engagement	Target antigen expression (PSMA, KLK2, STEAP2)	Acapatamab, pasotuxizumab, CB307	Phase I/II	Experimental	Cytokine release syndrome; antigen heterogeneity; immunosuppressive microenvironment; short response durability
CAR-T cell therapy	Engineered T cells targeting prostate-associated antigens	Target antigen expression (PSMA, PSCA, STEAP1)	P-PSMA-101; TGFβ-insensitive armored CAR-T	Phase I	Highly experimental	Limited persistence; T-cell exhaustion; CRS; manufacturing complexity and cost
Immune checkpoint inhibitors (combination-based)	Release of immune checkpoint blockade; synergy with DNA-damaging agents	MSI-H/dMMR, high TMB (rare in mCRPC)	Pembrolizumab-based combinations	Phase II/III (combinations)	Restricted to biomarker-selected subgroups	Immunologically cold microenvironment; minimal single-agent efficacy
Antibody-drug conjugates	Antibody-targeted delivery of cytotoxic payloads with bystander killing	Target antigen expression (B7-H3, TROP2, HER2, PSMA)	Ifinatamab deruxtecan, DB-1311/BNT324, ARX517	Phase I/II	Experimental	Target expression variability; payload-related toxicity; optimal sequencing
Steroidogenesis inhibitors (CYP11A1)	Blockade of upstream androgen biosynthesis	AR LBD mutations	Opevesostat (MK-5684)	Phase III ongoing	Late-stage development	Management of adrenal insufficiency; differentiation from established agents

*Clinical stage denotes the most advanced trial phase reached by any agent within the class as of the data cutoff date (March 27, 2026); it reflects the leading edge of development rather than the typical maturity of all trials in that class.

Several previous reviews have examined the treatment landscape of mCRPC, and the present analysis differs from and extends these studies in scope, methodology, and time frame. Leaning et al. performed systematic and structured literature reviews of published evidence to characterize treatment patterns, efficacy, safety, and the burden of disease in mCRPC (79), while Turco et al. reviewed the CRPC treatment landscape with an emphasis on patient selection and unmet clinical needs (80). Both works synthesized evidence from studies published at the time and therefore primarily reflect where the clinical evidence stood, rather than where research activity is heading. In contrast, the present study provides a registry-level analysis of 474 interventional trials registered in Trialtrove between January 2020 and March 2026, mapping ongoing and planned research activity in near-real time. This approach captures emerging therapeutic classes that were minimally represented in earlier landscape reviews, including next-generation radioligand therapies beyond ^177^Lu-PSMA, AR-directed PROTAC degraders, trispecific antibodies, novel ADCs, and CYP11A1 inhibitors, and allows trial-activity trends and treatment-sequencing-relevant combinations to be identified before their outcomes are published.

This analysis has several limitations. First, it draws solely on registered clinical trial data; some included trials may have been withdrawn or never published, while other active studies may not yet appear in registries. Second, because the data represent a snapshot taken in March 2026, trials initiated after the cutoff are not captured, and the status of ongoing trials may already have changed. Third, assigning each trial to a single therapeutic category inevitably oversimplifies complex combination regimens. Fourth, the distribution of trial phases and modalities does not directly correlate with clinical efficacy; many early-phase trials will fail to translate into practice-changing results. Fifth, our stratification of therapeutic classes by clinical maturity is a qualitative judgment based on the highest registered trial phase and regulatory status as of March 2026, and is therefore time-sensitive and subject to change as the field evolves. Finally, trial counts in a registry-based analysis reflect research activity rather than clinical maturity or therapeutic superiority; accordingly, statements on biological rationale and clinical positioning in this manuscript are derived from the published literature and should be distinguished from findings arising directly from the Trialtrove analysis.

## 5 Conclusion

In conclusion, based on the comprehensive analysis of the 474 clinical trials registered between January 2020 and March 2026, the therapeutic landscape for mCRPC has evolved into a highly diversified and dynamic field, overwhelmingly dominated by early-phase investigations. Targeted therapy, particularly PARP inhibitors, remain the leading class, while RPT, centered on ^177^Lu-PSMA, has reached a mature stage of development. Immunotherapy is expanding beyond checkpoint blockade into bispecific T-cell engagers and cellular therapies, and endocrine therapy is being reshaped by PROTAC degraders. ADCs are also gaining momentum, with B7-H3 as the most actively pursued targets. Critically appraised, the field can be stratified into approaches that have already entered routine practice (PARP inhibitors in HRR-altered disease and ^177^Lu-PSMA radioligand therapy), strategies in late-stage development (next-generation steroidogenesis inhibitors and selected ADCs), and modalities that remain exploratory (AR-directed PROTACs, T-cell engagers, and CAR-T cells). Realizing the promise of these emerging modalities will depend on biomarker-guided patient selection, rational mechanism-based combinations, and the resolution of class-specific translational barriers, including target heterogeneity, acquired resistance, and toxicity management.
